# Supersulfides regulate cell migration in human skin keratinocytes

**DOI:** 10.1247/csf.25018

**Published:** 2025-07-30

**Authors:** Kento Kunihiro, Katsura Sano

**Affiliations:** 1 ALBION Co., Ltd. 1-7-10 Ginza, Chuo-ku, Tokyo 104-0061, Japan

**Keywords:** supersulfide, cysteinyl-tRNA synthetase 2, keratinocyte, cell migration, wound healing

## Abstract

As the outermost organ, the skin is particularly susceptible to physical damage. Keratinocytes are a major component of the epidermis, and their migration plays a crucial role in skin wound healing. Supersulfides contribute to energy production to sustain the life activities of organisms and are anticipated to play a role in various physiological functions; however, minimal studies have investigated their presence and functions in the skin. This study aimed to determine the presence of supersulfides in the skin and investigate their effect on keratinocyte migration. Using sulfane sulfur probe 4 (SSP4), a fluorescent probe that detects sulfane sulfur, the presence of supersulfides in both skin tissue and keratinocytes was revealed. Moreover, the primary supersulfide biosynthetic enzyme, cysteinyl-tRNA synthetase 2 (CARS2), was expressed at both the tissue and cellular levels. CARS2 expression and SSP4 fluorescence intensity in keratinocytes increased during wound healing, suggesting that supersulfide is involved in the regulation of cell migration. Knockdown of CARS2 suppressed keratinocyte migration and markedly downregulated gene expression of various chemokines. Protein expression analysis revealed that supersulfides regulate E-cadherin and matrix metalloproteinase (MMP)-9 via extracellular signal-regulated kinase (ERK) and protein kinase B (Akt). Furthermore, Na_2_S_4_ treatment of keratinocytes with CARS2 knockdown restored cell migration. We propose that supersulfide in the skin represents a novel mechanism of re-epithelialization and may serve as a therapeutic target for skin wounds.

## Graphical Abstract

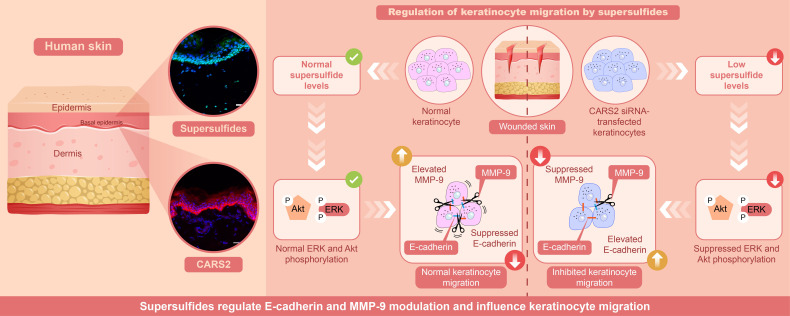

## Introduction

Supersulfides are sulfur species made up of chained sulfur atoms and include hydropersulfide and polysulfide species (Kasamatsu *et al.*, 2023a; Matsunaga *et al.*, 2023). Supersulfides function as potent redox signaling molecules. Redox reactions convert chemical stimulation into biological responses and regulate fundamental cellular activity processes, including energy metabolism, oxidative stress management, signal transduction, and cell development (Barayeu *et al.*, 2023). Prior to the advent of oxygen in the atmosphere, the sulfur elements that constitute supersulfides were employed as electron acceptors for energy production (Ogata *et al.*, 2023). Thus, sulfur metabolites have historically played a key role in the maintenance of life. Nonetheless, recent advances in analytical techniques have revealed that supersulfides (such as cysteine hydropersulfide and glutathione hydropersulfide) are universal bioactive metabolites produced by various organisms (Zhang *et al.*, 2024). Notably, supersulfides have been revealed to play an essential role in mitochondrial energy metabolism (Akaike *et al.*, 2017; Alam *et al.*, 2023). These findings indicate that sulfur respiration is functionally and evolutionarily conserved and produces energy via hybridization with oxygen respiration. Furthermore, cysteine residues in proteins have been demonstrated to form polysulfide bonds and alter their function (Kasamatsu *et al.*, 2023a; Matsunaga *et al.*, 2023).

Recently, several studies have reported the diverse functionalities of supersulfides, which include anti-inflammatory, antiviral, analgesic, gut microbiome modulation, innate immune regulation, anticancer, and anti-aging properties (Takeda *et al.*, 2023; Sun *et al.*, 2020; Matsunaga *et al.*, 2023; Yu *et al.*, 2022; Dillon *et al.*, 2021; Tsutsuki *et al.*, 2024). Particularly, supersulfides are known for their potent antioxidant activity, which is greater than that of ascorbic acid and Trolox (Kaneko *et al.*, 2022). Supersulfides are also recognized as key regulators of cellular redox homeostasis because they act as potent nucleophiles (Switzer, 2023). These supersulfides are abundant in the serum and widely distributed in other tissues and biological fluids, such as the heart, hair, and semen; however, there are limited reports on the detection of supersulfides in the skin (Ikeda *et al.*, 2019).

The skin is the largest organ of the human body and comprises three layers: the epidermis, dermis, and subcutaneous tissue. It has various functions, including protection from the external environment, thermoregulation, and sebum secretion. The skin is constantly exposed to stresses such as ultraviolet radiation, temperature changes, air pollution, chemical substances, and physical damage because it is the outermost layer of the organism (Harris and Grice, 2022). Keratinocytes are a major component of the epidermis and have protective barrier and wound-healing properties. Skin wound healing is an important physiological process that involves multiple cellular processes, including the proliferation, differentiation, and migration of keratinocytes (Shibata *et al.*, 2012). In addition, keratinocytes secrete various cytokines, chemokines, and growth factors to regulate the immune response of the skin through interactions with immune cells and maintain skin tissue homeostasis (Jiang *et al.*, 2020). Keratinocyte migration is the first step in re-epithelialization and is essential for optimal wound closure. Accordingly, inhibiting cell migration-associated signaling pathways can lead to chronic wounds, highlighting that keratinocyte migration plays a central role in facilitating skin wound healing. Cysteine tRNA synthetase 2 (CARS2) is the primary enzyme that produces cysteine persulfide *in vivo* (Abidin *et al.*, 2023). Previous studies have demonstrated that CARS2 deficiency significantly affects the migration of mouse GnRH-expressing neuronal cells (Wang *et al.*, 2015). Based on these findings, we hypothesized that supersulfides biosynthesized by CARS2 are present in the skin and regulate keratinocyte migration. Therefore, this study aimed to detect supersulfides in human skin tissue and keratinocytes using sulfane sulfur probe 4 (SSP4) and to evaluate the effect of supersulfides on cell migration through scratch assays, gene expression analysis, and protein expression analysis.

## Materials and Methods

### Antibodies

The following antibodies were used in this study: anti-CARS2 (cat. no. OAAB04900), purchased from Aviva Systems Biology (San Diego, CA, USA); anti-p38 (cat. no. 51115-1-AP), anti-phospho-p38 (p-p38) (cat. no. 28796-1-AP), and anti-E-cadherin antibodies (cat. no. 20874-1-AP), purchased from Proteintech (Chicago, IL, USA); anti-ERK (cat. no. #4695), anti-phospho-ERK (p-ERK) (cat. no. #4370), anti-Akt (cat. no. #9272), anti-phospho-Akt (p-Akt) (cat. no. #9271), anti-MMP-2 (cat. no. #40994), anti-MMP-9 (cat. no. #13667), anti- GAPDH (cat. no. #2118), and anti-rabbit horseradish peroxidase-linked antibodies (cat. no. #7074), purchased from Cell Signaling Technology (Danvers, MA, USA); and anti-MMP-10 (cat. no. ARG59021) antibody, obtained from Arigo Biolaboratories (Hsinchu, Taiwan).

### Cell culture and human skin tissue

Normal human epidermal keratinocytes (NHEKs) were purchased from Thermo Fisher Scientific (Waltham, MA, USA). The cells were cultured in EpiLife medium (Thermo Fisher Scientific) supplemented with 1% human keratinocyte growth supplement (HKGS; Thermo Fisher Scientific). Cells were grown at 37°C in a humidified incubator containing 5% CO_2_, according to the manufacturer’s instructions.

Human skin samples (25-year-old White donor, abdomen) were purchased from Biopredic International (Saint-Grégoire, France) through KAC (Kyoto, Japan). The human experiments were approved by the ethics committee of KAC for human tissue products.

### Detection of supersulfide by SSP4

Supersulfide in the skin tissue and keratinocytes was detected using SSP4 (Dojindo, Kumamoto, Japan), according to the manufacturer’s protocol. Briefly, human skin tissue was embedded in an Optimal Cutting Temperature (OCT) compound (Sakura Finetek Japan, Tokyo, Japan) and stored at –80°C. The skin samples were cut into 10 μm slices and mounted on glass slides. The skin samples were washed with phosphate-buffered saline (PBS), subsequently stained with 100 μM SSP4, 1 mM cetyltrimethylammonium bromide (CTAB), and 1 μg/mL DAPI (Dojindo), and incubated at 25°C for 1 h. The samples were examined under a BZ-X700 fluorescence microscope (Keyence, Osaka, Japan).

NHEK cells were seeded (1 × 10^4^ cells/well) in 96-well black plates and cultured overnight. The cultured cells were subsequently washed with Hanks’ balanced salt solution (HBSS) and fixed in 4% paraformaldehyde (PFA) at 25°C for 25 min. After washing, the cells were stained with 20 μM SSP4, 0.5 mM CTAB, and 1 μg/mL DAPI at 25°C for 30 min. Fluorescence images were captured using a BZ-X700 fluorescence microscope, and the fluorescence intensity (em/ex, 482/515 nm) was determined using a SpectraMax i3x (Molecular Devices, San Jose, CA, USA).

### Quantitative targeted supersulfide metabolomics

Cysteine hydropersulfide (CysSSH) and glutathione hydropersulfide (GSSH) in human skin tissue and keratinocytes were quantified using a modified version of a previously reported protocol (Kasamatsu *et al.*, 2021). Samples were homogenized by sonication (15 s × 3 cycles, 30 s intervals) in ice-cold 100 mM ammonium formate solution containing 1 mM N-iodoacetyl L-tyrosine methyl ester (TME-IAM) and 70% methanol, followed by incubation at 37°C for 1 h. After centrifugation, the supernatants were diluted 1:3 with 0.1% formic acid (FA) and spiked with 50 nM isotope-labeled TME-AM-adduct standards. Quantification was conducted via liquid chromatography–electrospray ionization–tandem mass spectrometry (LC–ESI–MS/MS) using a stable isotope-labeled standard dilution method. Results were normalized to protein content.

LC–ESI–MS/MS analyses were carried out using a Shimadzu LCMS-8060 triple quadrupole mass spectrometer (Shimadzu, Kyoto, Japan) with a Nexera X2 UHPLC system (Shimadzu) and a Mightysil-C18 column (50 × 2.0 mm, KANTO CHEMICAL Co., Tokyo, Japan). For TME-IAM adducts, separation was achieved using a methanol gradient: 1% B (0–1 min), 80–99% B (11–11.1 min), and 99% B (11.1–12.5 min). The mobile phase contained 0.1% FA, and the flow rate was set at 0.4 mL/min at 40°C. The MS was operated in positive mode (capillary voltage, 1,000 V; desolvation gas, 1,000 L/h; source temperature, 500°C). Multiple reaction monitoring parameters were set as previously reported (Kasamatsu *et al.*, 2021, 2023b).

### Immunofluorescence staining

NHEK cells were seeded (2 × 10^4^ cells/well) in a 4-well chamber slide and cultured overnight at 37°C. The cells were fixed with 4% PFA in PBS for 15 min at 25°C. Human skin tissue was embedded in the OCT compound, and skin sections were prepared as described above. Fixed cells and skin section samples were washed with PBS, permeabilized with PBS containing 0.1% Triton X-100 (Nacalai Tesque, Kyoto, Japan) for 20 min at 25°C, and treated with Blocking One Histo solution (Nacalai Tesque) for 10 min at 25°C. After washing with PBS, the samples were incubated for 24 h at 4°C with anti-CARS2 antibody in Blocking One Histo solution (1:200 dilution) and visualized using Alexa Fluor 488 (1:1,000 dilution; Abcam, Cambridge, UK) and DAPI (1:1,000 dilution) in the dark for 1 h at 25°C. After washing, the samples were mounted in ProLong^TM^ Diamond Antifade Mountant (Thermo Fisher Scientific), and fluorescence images were obtained using a BZ-X700 fluorescence microscope.

### Small interfering RNA (siRNA) transfection

CARS2 knockdown in NHEK cells was performed using Lipofectamine RNAiMAX Reagent (Thermo Fisher Scientific) according to the manufacturer’s instructions. Briefly, NHEK cells were seeded (2.5 × 10^5^ cells/well) in 6-well plates and cultured overnight. The cells were transfected with 50 nM scrambled siRNA (scRNA; Thermo Fisher Scientific, cat. no. 4390846) or CARS2 siRNA (siCARS2; Thermo Fisher Scientific, siRNA ID s35866) and incubated for 24–48 h at 37°C. The efficiency of CARS2 knockdown was evaluated using quantitative PCR (qPCR) and Western blotting. Furthermore, the effect of knockdown on supersulfide biosynthesis was examined using SSP4.

### Mitochondrial membrane potential assay

Mitochondrial membrane potential was measured using the JC-1 MitoMP Detection Kit (Dojindo) according to the manufacturer’s instructions. NHEK cells were seeded (1 × 10^4^ cells/well) in 96-well black plates and cultured overnight. The cells were transfected with scRNA or CARS2 siRNA and incubated for 24 h. JC-1 dye was then added to the cells at a final concentration of 2 μmol/L and incubated at 37°C for 1 h. After washing with HBSS, imaging buffer solution was added to the cells. Fluorescence images were captured using the BZ-X700 fluorescence microscope, and the fluorescence intensity (em/ex, 490/525 nm and 540/590 nm) was determined using the SpectraMax i3x microplate reader.

### Cell migration assay

NHEK cells were seeded (1 × 10^5^ cells/well) in 24-well plates and cultured overnight. The cells were transfected with scRNA or CARS2 siRNA for 24 h. The following day, the transfected cells were scratched using a sterile 200 μL pipette tip. After washing with PBS, medium or Na_2_S_4_ solution was added to the cells. Each well was examined via time-lapse microscopy using a BZ-X700 fluorescence microscope. Data were calculated as the percentage of the initial (0 h) and final (20 h) scratch areas relative to scRNA-transfected cells.

### Cell viability assay

Cell viability was measured using the Calcein-AM assay. NHEK cells were seeded (1 × 10^4^ cells/well) in 96-well black plates and cultured overnight. The cells were treated with either scRNA, CARS2 siRNA, or Na_2_S_4_, and then stained with 10 mM Calcein-AM (Dojindo) at 37°C for 30 min. The fluorescence intensity (em/ex, 485/530 nm) of each well was measured using a SpectraMaxs i3x. Data were calculated as relative cell viability compared with normal cells.

### qPCR assay

Total RNA was extracted using the TRI reagent (Molecular Research Center, Cincinnati, OH, USA) according to the manufacturer’s instructions. cDNA was synthesized via reverse transcription using the PrimeScript RT reagent kit (Takara Bio, Shiga, Japan). Total mRNA was quantified using a NanoDrop spectrophotometer (Thermo Fisher Scientific). qPCR was performed using a LightCycler 96 PCR system (Roche, Basel, Switzerland) and Luna Universal qPCR Master Mix (New England Biolabs, Ipswich, MA, USA). The target genes and corresponding primer sequences used were as follows: CARS2: forward primer (F), 5'-GAAGCCGCCTCCTGGTATAG-3'; reverse primer (R), 5'-TGCTGCATCCAAAAACCTTGG-3', GAPDH: forward primer (F), 5'-GAGCCACATCGCTCAGACAC-3'; reverse primer (R), 5'-TTGCCATGGGTGGAATCATA-3', CXCL1: forward primer (F), 5'-AGGCAGGGGAATGTATGTGC-3'; reverse primer (R), 5'-GCCCCTTTGTTCTAAGCCAG-3', CXCL2: forward primer (F), 5'-ACCTGGATTGCGCCTAATGT-3'; reverse primer (R), 5'-ATGGGAGAGTGTGCAAGTAGA-3', CXCL5: forward primer (F), 5'-GGACCAGAGAGAGCTTGGAA-3'; reverse primer (R), 5'-CCAGCGTGGCCACCTTATAT-3', and CXCL8: forward primer (F), 5'-GCTCTGTGTGAAGGTGCAGT-3'; reverse primer (R), 5'-TTCTCCACAACCCTCTGCAC-3'. The measured data were analyzed using the delta threshold cycle method, and the expression of each gene was normalized to that of GAPDH, as described previously (Sano *et al.*, 2014).

### Western blotting analysis

The cells were washed with PBS and collected using RIPA buffer (Nacalai Tesque) containing a protease inhibitor cocktail (Nacalai Tesque). The cells were centrifuged at 20,000 × *g* for 30 min at 4°C to extract proteins. The supernatant was mixed with Sample Buffer Solution with 2-ME (2x) (Nacalai Tesque), boiled at 100°C for 5 min, and stored at –30°C. Samples were separated using 10% SDS-PAGE and transferred onto nitrocellulose membranes (Bio-Rad, Hercules, CA, USA). The membranes were then treated with Blocking One or Blocking One-P solution (Nacalai Tesque) for 1 h at 25°C. After washing with Tris-buffered saline (TBS) containing 0.1% Tween-20 (TBST), the samples were incubated overnight with primary antibodies diluted in Can Get Signal Solution 1 (TOYOBO, Osaka, Japan) at 4°C (namely anti-CARS2, anti-p38, anti-p-p38, anti-ERK, anti-p-ERK, anti-Akt, anti-p-Akt, anti-E-cadherin, anti-MMP-2, anti-MMP-9, anti-MMP-10, and anti-GAPDH; 1:1,000 dilution). The following day, the membranes were washed with TBST and incubated with a secondary antibody diluted in Can Get Signal Solution 2 (TOYOBO) for 1 h at 37°C (anti-rabbit HRP-linked antibody; 1:1,000 dilution). Samples were analyzed using a LuminoGraph I system (Atto, Tokyo, Japan).

### Statistical analysis

All statistical analyses were performed using GraphPad Prism version 9 for Windows (GraphPad Software, San Diego, CA, USA). Values are reported as the mean ± standard deviation (SD). Statistical significance was determined using the Student’s t-test.

## Results

### Detection of supersulfide and CARS2 expression in human skin tissue and keratinocytes

**Fig. 1:**
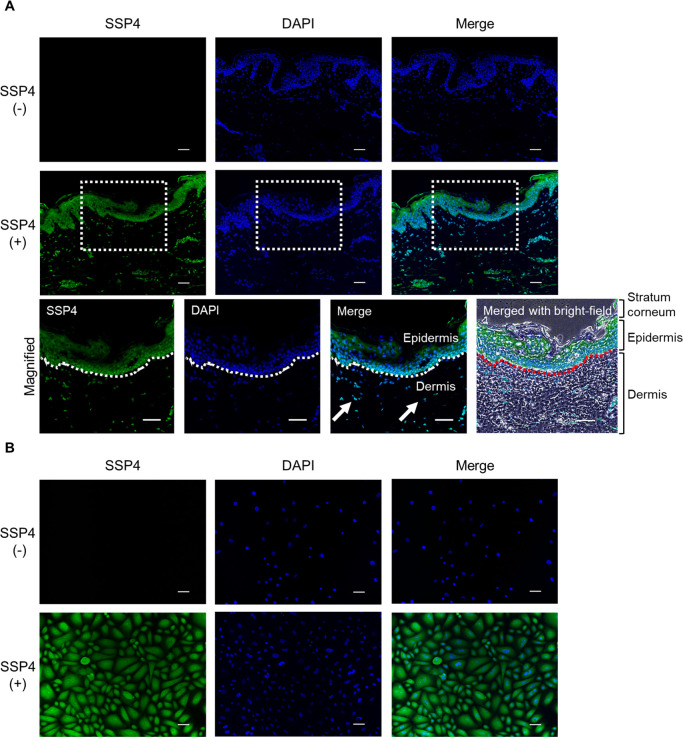
Detection of supersulfide in human skin tissue and keratinocytes Supersulfides and nuclei were stained with sulfane sulfur probe 4 (SSP4) (green) and DAPI (blue), respectively. (A) Fluorescence imaging of supersulfide in skin tissue. The white arrows indicate supersulfides in the dermis. (B) Fluorescence imaging of supersulfide in human skin keratinocytes. Scale bars represent 50 μm.

First, we used SSP4 to detect supersulfide in human skin with reference to previous studies (Takahashi *et al.*, 2021). The stained images showed the green fluorescence of SSP4 in human skin tissue, indicating the presence of supersulfide in the skin (Fig. 1A). Moreover, supersulfide was detected in both the epidermis and dermis. We subsequently measured the intracellular supersulfide levels in keratinocytes. Fig. 1B shows the fluorescence intensity of SSP4 in keratinocytes. Furthermore, LC–ESI–MS/MS analysis detected supersulfides such as cysteine hydropersulfide (CysSSH) and glutathione hydropersulfide (GSSH) in human skin tissue and keratinocytes (Table S1).

**Fig. 2:**
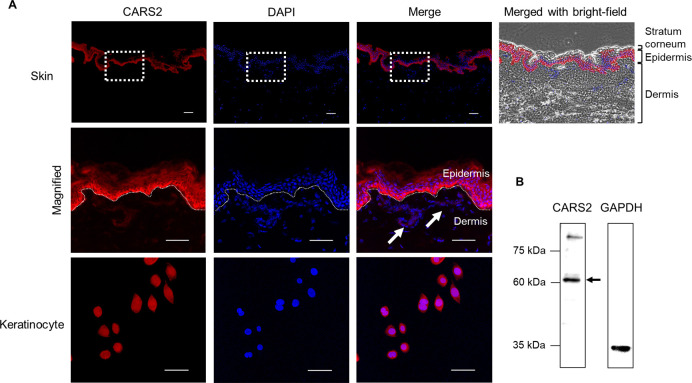
Expression of cysteinyl-tRNA synthetase 2 (CARS2) in human skin tissue and keratinocytes (A) Immunocytochemical staining of CARS2 antibody (red) and DAPI (blue). Scale bar represents 50 μm. The white arrows indicate CARS2 expression in the dermis. (B) CARS2 protein expression in human skin keratinocytes determined by Western blot analysis.

CARS2 plays a key role in endogenous supersulfide production, indicating that these enzymes function in major biosynthetic pathways for supersulfides (Akaike *et al.*, 2017). Therefore, we determined the expression and localization of CARS2 in human skin tissues and keratinocytes via immunostaining (Fig. 2A), which revealed that CARS2 was localized in the basal cells of the epidermis, with minimal expression in the stratum corneum and dermis. This was further confirmed with Western blotting analysis, which identified CARS2 as an approximately 62 kDa band in human skin keratinocytes (Fig. 2B).

### Supersulfide levels and CARS2 expression during wound healing assay in human skin keratinocytes

**Fig. 3:**
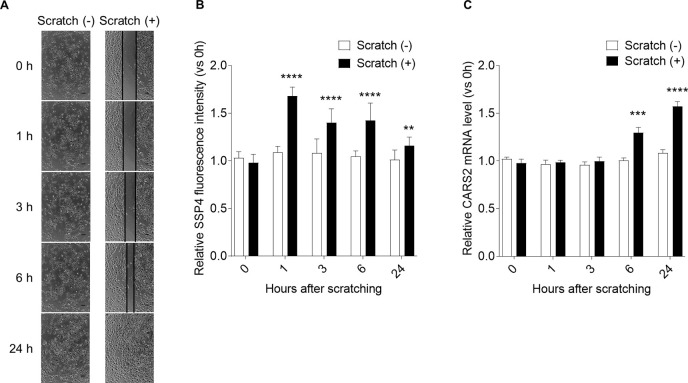
Supersulfide detection and CARS2 expression during wound healing assay in human skin keratinocytes Human skin kerationocytes were cultured to 80–90% confluence and then scratched using a sterile 200 μL pipette tip. After washing with PBS, the cells were incubated with fresh medium at 37°C for 1–24 h. Supersulfide levels and CARS2 expression were confirmed by SSP4 staining and qPCR at each time point, including the initial time point (0 h). Data were presented as relative values to the 0 h value for both scratched and non-scratched cell groups. (A) Images of keratinocyte scratch wound closure over time. Scale bars represent 100 μm. (B) The fluorescence intensity of SSP4 over time during wound healing assay was measured using a fluorescence plate reader. (C) mRNA expression levels of CARS2 were normalized to GAPDH expression. The data represent mean ± SD (^**^p<0.01 and ****p<0.0001 vs 0 h).

To investigate the relationship between supersulfides and keratinocyte migration, we examined supersulfide levels and CARS2 expression during wound healing assay. The cells were scratched with a sterile 200 μL pipette tip and examined for SSP4 fluorescence intensity and CARS2 gene expression at 0, 1, 3, 6, and 24 h. Cells began to fill the wound after 6 h of scratching, and the wound was completely closed after 24 h (Fig. 3A). The fluorescence intensity of SSP4 increased significantly after cell scratching (Fig. 3B). No change over time was observed in the fluorescence intensity of SSP4 in the non-scratched control cell group (Fig. 3B). Gene expression levels of CARS2 were also significantly increased during the wound healing assay (Fig. 3C).

### CARS2 knockdown in human skin keratinocytes

**Fig. 4:**
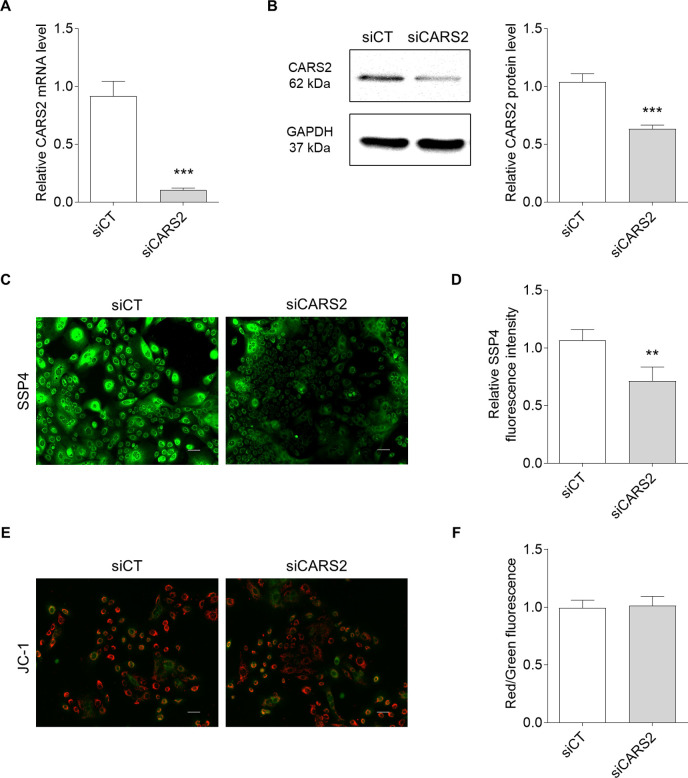
Effect of CARS2 knockdown in human skin keratinocytes (A) Gene expression of CARS2 in human skin keratinocytes. Total RNA was extracted from keratinocytes, and CARS2 mRNA levels were determined using qPCR. (B) Protein expression of CARS2 in human skin keratinocytes was determined by Western blotting analysis. Fluorescence imaging (C) and intensity (D) of supersulfides in human skin keratinocytes stained with SSP4. Fluorescence imaging (E) and the relative red to green fluorescence ratio (F) of mitochondrial membrane potential in human skin keratinocytes were determined using the JC-1 MitoMP Detection Kit. Scale bar represents 50 μm. The data represent the mean ± SD (**p<0.01 and ***p<0.001 vs. siCT).

To investigate the effects of supersulfide on keratinocyte migration, we generated CARS2-depleted keratinocytes. The efficiency of CARS2 knockdown was examined using qPCR and Western blot analysis. The gene expression of CARS2 in keratinocytes with CARS2 knockdown decreased by approximately 90% compared to that in scRNA-transfected cells (Fig. 4A). Similarly, the protein expression of CARS2 was also downregulated in CARS2 knockdown cells (Fig. 4B). To determine the effects of CARS2 silencing on supersulfide biosynthesis, we measured SSP4 fluorescence intensity in keratinocytes transfected with scRNA and CARS2 siRNA. The stained images and fluorescence intensities revealed that the green fluorescence of SSP4 was significantly reduced in CARS2 siRNA-transfected keratinocytes, indicating that CARS2 knockdown suppressed supersulfide production (Fig. 4C and D). Therefore, these results demonstrated that CARS2 knockdown successfully suppressed the capacity of keratinocytes to biosynthesize supersulfides. Furthermore, we evaluated the effect of CARS2 siRNA on mitochondrial function and cytotoxicity using the JC-1 and Calcein-AM assays. No significant difference was observed in the mitochondrial membrane potential (Fig. 4E and F) and cell viability (Fig. S1) of CARS2 siRNA-transfected keratinocytes compared to that of control cells.

### Cell migration in CARS2-depleted keratinocytes

**Fig. 5:**
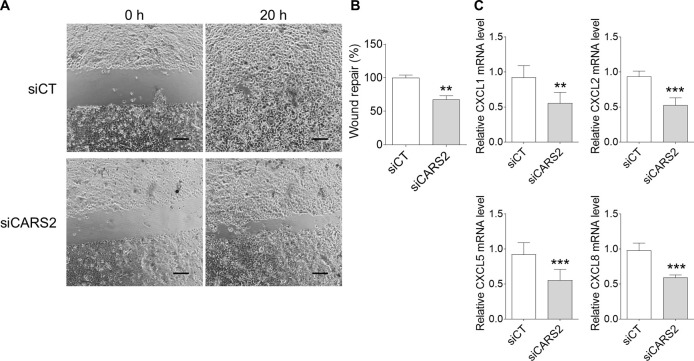
Effect of CARS2 knockdown on cell migration and gene expression of C-X-C motif chemokine ligands (CXCLs) in human skin keratinocytes A cell migration assay was performed following 24 h of transfection with scRNA or CARS2 siRNA. Time-lapse photographs were taken every hour for 20 h after scratching. (A) Images of the cell migration assay at 0 and 20 h post-scratching. Scale bar represents 200 μm. (B) Quantification of the percentage of wound repair. (C) mRNA expression levels of CXCLs were normalized to GAPDH expression. Data represent the mean ± SD (**p<0.01 and ***p<0.001 vs. siCT).

Keratinocyte migration determines the rate of skin re-epithelialization (Wu *et al.*, 2016). We performed a scratch assay to evaluate the effect of CARS2 knockdown on keratinocyte migration. Keratinocytes were transfected with scRNA or CARS2 siRNA for 24 h prior to the scratch assay and subsequently observed every hour via time-lapse microscopy. The results indicated that scRNA-treated keratinocytes migrated to approximately 95% of the scratched area, whereas CARS2 siRNA-transfected keratinocytes migrated to only 60% of the scratched area 20 h after scratching (Fig. 5A and B). Thus, CARS2 knockdown significantly suppressed keratinocyte migration.

Various chemokines (e.g., CXCL1 and CXCL8) have been reported to play important roles in keratinocyte migration during skin wound healing (Piipponen *et al.*, 2020). In this study, we investigated the effects of CARS2 knockdown on the expression of several chemokine genes in keratinocytes. We revealed that the gene expression levels of CXCL1, CXCL2, CXCL5, and CXCL8 were significantly downregulated in CARS2 siRNA-transfected keratinocytes (Fig. 5C). These results indicate that suppressing CARS2 expression inhibits keratinocyte chemotaxis.

### Signal pathway analysis of CARS2-depleted keratinocytes

**Fig. 6:**
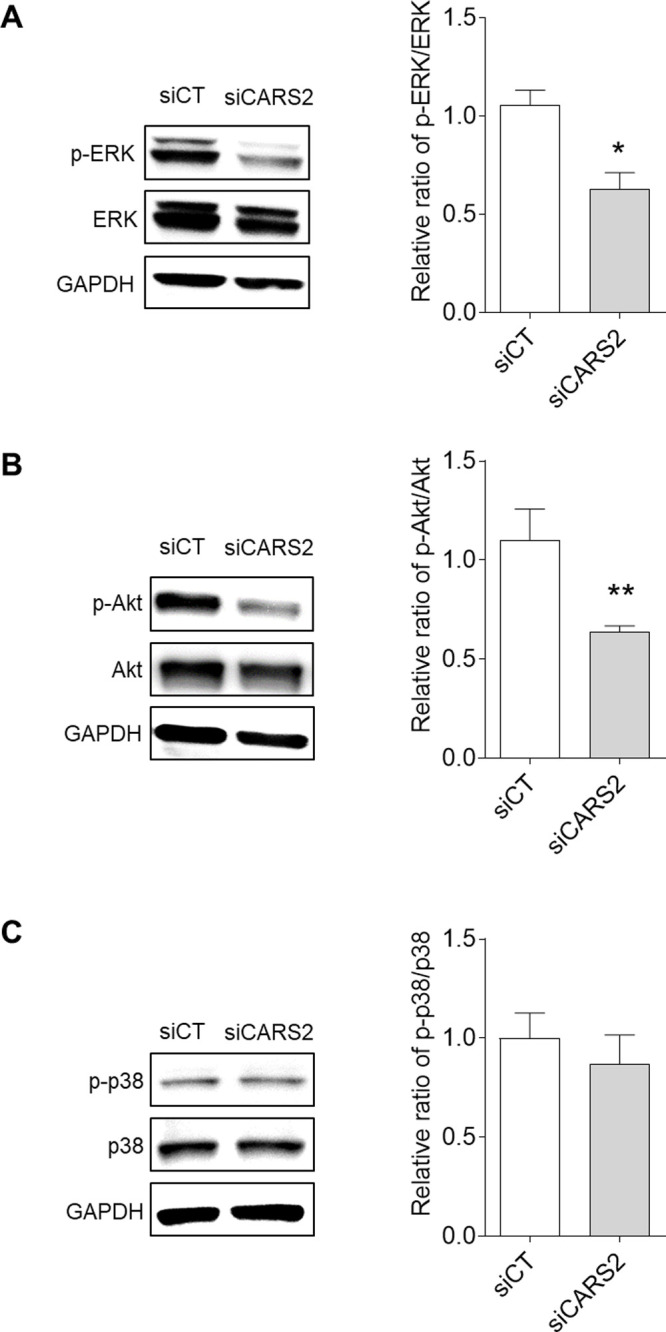
Evaluation of the expression of signaling pathway proteins and their corresponding phosphorylated forms after CARS2 knockdown in human skin keratinocytes Phosphorylation of total extracellular signal-regulated kinase (ERK) (A), protein kinase B (Akt) (B), and p38 (C) was determined using Western blot analysis. Data represent the mean ± SD (*p<0.05 and **p<0.01 vs. siCT).

**Fig. 7:**
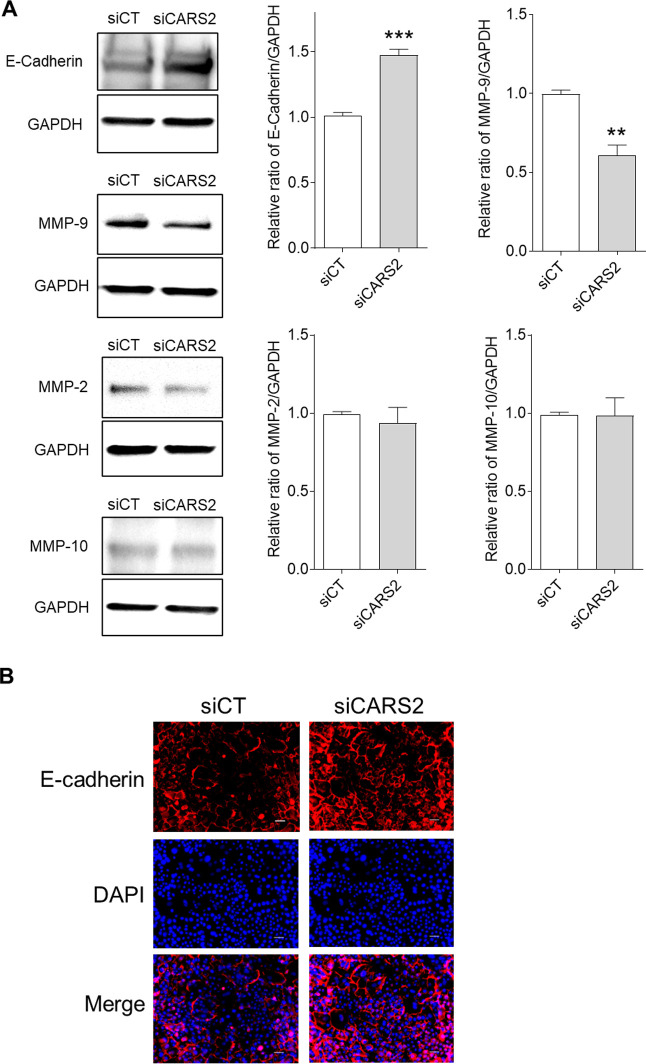
Expression of E-cadherin and matrix metalloproteinases (MMPs) after CARS2 knockdown in human skin keratinocytes (A) Protein expression of E-cadherin and MMPs was determined by Western blot analysis. Data represent the mean ± SD (**p<0.01 and ***p<0.001 vs. siCT). (B) Immunohistochemistry analysis of E-cadherin expression in keratinocytes transfected with scRNA or CARS2 siRNA. Scale bar represents 50 μm.

To elucidate the mechanisms underlying the suppression of cell migration induced by CARS2 knockdown, we examined the expression of several associated signaling molecules and their corresponding phosphorylated forms in keratinocytes transfected with scRNA or CARS2 siRNA. Western blot analysis showed that the phosphorylation levels of ERK and Akt decreased in CARS2-knockdown keratinocytes (Fig. 6A and B). In contrast, p38 phosphorylation did not change significantly (Fig. 6C). Furthermore, keratinocytes with CARS2 knockdown exhibited increased E-cadherin expression (Fig. 7A and B) and significantly decreased MMP-9 expression (Fig. 7A). In contrast, MMP-2 and MMP-10 expression remained unchanged in CARS2-knockdown keratinocytes (Fig. 7A).

### Regulation of keratinocyte migration by CARS2-mediated supersulfide biosynthesis

**Fig. 8:**
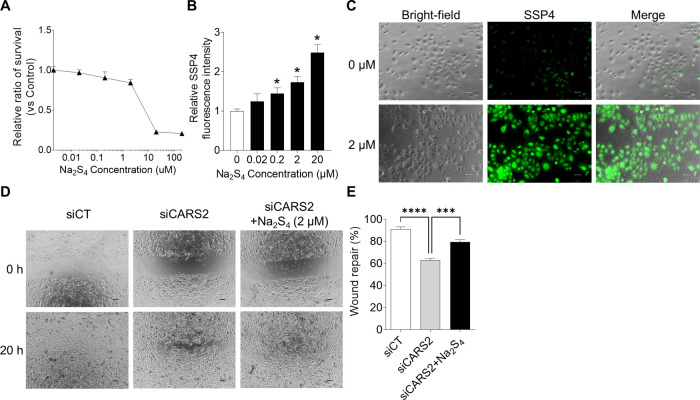
Effect of Na_2_S_4_ on cell migration in CARS2 siRNA-transfected keratinocytes (A) Cell viability was assessed by Calcein-AM assay and calculated relative to that of normal cells. (B, C) Supersulfides were stained with SSP4 (green). Fluorescence intensity (B) and imaging (C) in Na_2_S_4_-treated cells are shown. Data represent mean ± SD (*p<0.05 vs. 0 μM Na_2_S_4_). Scale bar represents 50 μm. Keratinocytes were transfected with scRNA or CARS2 siRNA. Na_2_S_4_ was then added to the cells, and the scratch assay was performed. Images were taken by time-lapse every hour for 20 h after scratching. (D) Images of cell migration assay at 0 and 20 h. Scale bar represents 200 μm. (E) Quantification of the percentage of wound repair. Data represent mean ± SD (***p<0.001 and ****p<0.0001).

To determine whether supersulfides are essential for CARS2-mediated regulation of keratinocyte migration, we performed rescue experiments in which CARS2-knockdown cells were supplemented with Na_2_S_4_. First, cell viability and supersulfide levels were measured in Na_2_S_4_-treated keratinocytes. The results showed that Na_2_S_4_ was not cytotoxic at the low concentration range (Fig. 8A). The fluorescence intensity of SSP4 increased in a concentration-dependent manner in cells treated with Na_2_S_4_ (Fig. 8B). In addition, intracellular supersulfide levels were significantly increased by Na_2_S_4_ treatment as observed in fluorescence and bright-field imaging of supersulfides (Fig. 8C). Next, we examined whether the suppression of cell migration capacity in keratinocytes with CARS2 knockdown could be restored by Na_2_S_4_ supplementation. As shown in Fig. 8D and E, Na_2_S_4_ treatment rescued cell migration in CARS2 knockdown cells. On the other hand, addition of Na_2_S_4_ to normal keratinocytes did not significantly affect cell migration (Fig. S2).

## Discussion

Long-chain sulfur compounds (supersulfides) are present in both prokaryotes and eukaryotes, including mammals such as mice and humans (Toohey, 2011; Fukuto *et al.*, 2012; Numakura *et al.*, 2017; Dóka *et al.*, 2016). These supersulfides play important roles in regulating mitochondrial biosynthesis and cellular regulatory processes (Akaike *et al.*, 2017). However, only a few studies have detected the presence of supersulfides in human skin, and their physiological functions remain unexplored. In this study, we proposed that supersulfides regulate keratinocyte migration.

In a recent study, supersulfide was detected in biological fluids, such as serum, tear fluid, saliva, semen, and nasal secretions, and was implicated in diverse physiological functions (Ikeda *et al.*, 2019). Dermal fibroblasts in the skin contain supersulfides, whose levels have been shown to decrease with age (Zivanovic *et al.*, 2019). Our results showed that supersulfides were present in both the epidermis and dermis. In addition, supersulfides were detected in keratinocytes. These results indicate that a biosynthetic pathway for supersulfide exists in the skin. Cystathione γ-lyase (CSE), cystathione-β-synthase (CBS), 3-mercaptopyruvate sulfurtransferase (3-MST), and CARS2 are biosynthetic enzymes responsible for synthesizing supersulfides (Tsutsuki *et al.*, 2024; Goren *et al.*, 2019; Takematsu *et al.*, 2020). Although previous studies have not identified CSE, CBS, and 3-MST as the main pathways for supersulfide biosynthesis in mammals, CARS2 was recently recognized as the key enzyme for endogenous supersulfide production (Abidin *et al.*, 2023). Hence, we investigated the expression and localization of CARS2 in human skin tissue and keratinocytes. Our findings confirmed the presence of CARS2 in both human skin tissues and keratinocytes (Fig. 2). Notably, CARS2 was localized predominantly in epidermal basal cells, suggesting that human skin supersulfide may be biosynthesized via CARS2 in keratinocytes.

Previous studies have reported the involvement of CARS2 in cell migration (Wang *et al.*, 2015). We found that supersulfide levels and CARS2 expression were significantly increased during scratch-induced cell wounding (Fig. 3). These results suggest that supersulfides in keratinocytes may play an important role in the wound healing process. Furthermore, our results showed that CARS2 knockdown significantly suppressed keratinocyte migration (Fig. 5). Knockdown of CARS2 had no significant effect on mitochondrial membrane potential or cell viability, indicating that the inhibition of keratinocyte migration was not due to changes in mitochondrial function or cell proliferation (Fig. 4E, F). Keratinocyte-derived chemokines (such as CXCL1, CXCL5, and CXCL8) contribute to cell migration and play important roles in the recruitment and activation of neutrophils and macrophages at wound sites (Jiang *et al.*, 2020; Piipponen *et al.*, 2020). In this study, keratinocytes with knockdown of CARS2 showed decreased expression of various chemokines (Fig. 5). This result also supports the involvement of keratinocyte supersulfides in the skin wound healing process.

During the wound healing process, epidermal basal cells proliferate and differentiate to form a stratified epithelium. This process contributes to restoration of the barrier function. As shown in Fig. 2, CARS2, a supersulfide synthase, is strongly expressed in the epidermal basal cell layer, while it is weakly expressed in the stratum corneum. Since keratinocytes in the epidermal basement membrane primarily replicate and migrate to regenerate the skin, high expression of CARS2 in epidermal basal cells may be important for increasing intracellular supersulfide levels and promoting wound healing. On the other hand, since the stratum corneum is composed of enucleated cells derived from keratinocytes, weak expression of CARS2 indicates a low need for supersulfide production. In light of the above, these findings suggest that CARS2-mediated supersulfide biosynthesis in epidermal basal cells, which play a central role in the skin repair process, may regulate cell migration.

The ERK, p38 mitogen-activated protein kinase (MAPK), and phosphatidylinositol-3 kinase (PI3K)/Akt pathways are important signaling molecules involved in skin wound healing (Beserra *et al.*, 2018). ERKs (ERK1 and ERK2) are protein-serine/threonine kinases associated with the Ras-Raf-MEK-ERK signaling pathway that modulate cell migration, cell adhesion, cell proliferation, cell differentiation, transcription, and metabolism (Roskoski, 2012). Activation of the p38 MAPK signaling pathway has been reported to induce the migration of keratinocytes to injury sites and cell proliferation processes (Loughlin and Artlett, 2011). The PI3K/Akt pathway has been demonstrated to influence migration speed and promote the migration of different types of cells, including keratinocytes and fibroblasts (Sasaki *et al.*, 2004). In this study, we showed that CARS2 knockdown significantly suppressed ERK and Akt activation (Fig. 6). In a previous study, glutathione trisulfide was reported to activate the ERK signaling pathway and regulate inflammation-related genes (Tawarayama *et al.*, 2022). Na_2_S_4_ has been reported to reduce oxidative damage and exhibit cytoprotective effects via activation of the Akt pathway (Koike *et al.*, 2013). Thus, it is considered that supersulfides regulate various cellular functions through the ERK and Akt signaling pathways. The ERK and Akt signaling pathways are known to regulate the levels of cytokines and chemokines in keratinocytes and control cell migration (Mi *et al.*, 2018). Our findings suggest that supersulfides in keratinocytes may contribute to wound healing via activation of the ERK and Akt pathways. E-cadherin and MMPs are also important factors in keratinocyte migration (Wu *et al.*, 2016). E-cadherin is a transmembrane glycoprotein that promotes cell-cell adhesion and is essential for maintaining the balance between cell migration and adhesion (Asai *et al.*, 2016). MMPs are a family of enzymes that degrade cell adhesion molecules (e.g., cadherins and integrins) and regulate cell migration (Ray *et al.*, 2023; Lai *et al.*, 2017). MMP-2, MMP-9, and MMP-10 contribute to keratinocyte migration (Xue and Jackson, 2008; Schlage *et al.*, 2015). Our findings showed that CARS2 knockdown in keratinocytes increased E-cadherin expression and decreased MMP-9 expression. Furthermore, the Akt and ERK signaling pathways are involved in the expression of E-cadherin and MMPs and play key roles in cell migration (Xu *et al.*, 2020; Jin *et al.*, 2021). Therefore, the inhibition of keratinocyte migration caused by the loss of CARS2 is mediated by the regulation of E-cadherin and MMP-9 expression via the ERK and Akt signaling pathways.

**Fig. 9:**
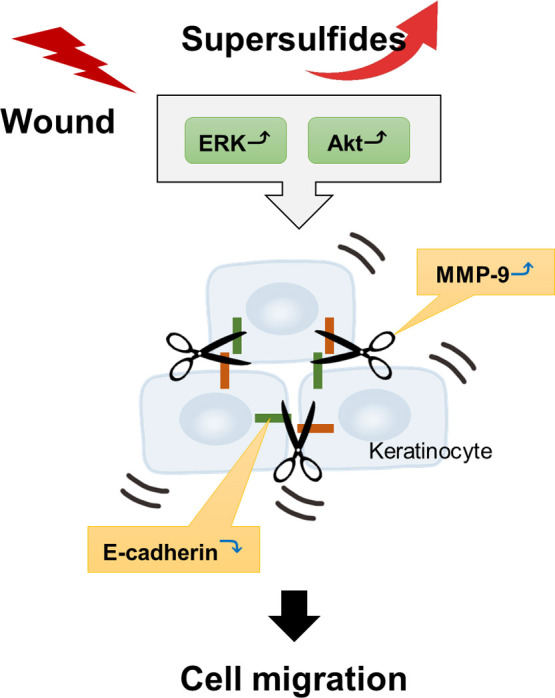
Hypothetical proposed model for the regulation of cell migration through the CARS2-mediated biosynthesis of supersulfide in keratinocytes Upon skin wounding, CARS2 activation leads to supersulfide production, which enhances cell migration through activation of ERK and Akt signaling pathways and modulation of E-cadherin and MMP-9 expression, ultimately promoting accelerated wound closure.

To clarify whether supersulfides regulate keratinocyte migration, we treated cells with Na_2_S_4_ and assessed its effect on cell migration ability (Fig. 8). Interestingly, Na_2_S_4_ treatment restored cell migration in keratinocytes with suppressed CARS2 expression, whereas treatment of normal keratinocytes with Na_2_S_4_ had little effect on cell migration. These findings indicate that intracellular supersulfide levels are appropriately regulated in normal skin and that supersulfides are biosynthesized along with CARS2 activation during wounding, contributing to accelerated wound healing. The hypothetical model for the regulation of cell migration by CARS2-mediated biosynthesis of supersulfide in keratinocytes is shown in Fig. 9. Based on our findings, we speculate that in damaged skin, cell migration is enhanced by activation of the ERK and Akt pathways in keratinocytes (Fig. 6) and by modulation of E-cadherin and MMP-9 expression (Fig. 7). However, the present study revealed a discrepancy between the fluorescence intensity of SSP4 and the timing of the increase in CARS2 expression in keratinocytes in the scratch assay (Fig. 3). This result suggests that supersulfides may have been produced temporarily due to the presence of a supersulfide synthesis pathway other than CARS2 or activation of existing CARS2, and that supersulfide was subsequently replenished or maintained by increasing CARS2 expression. Overexpression of CARS2, along with other sulfur-metabolizing enzymes, and *in vivo* studies are necessary to elucidate the regulatory mechanisms of supersulfide-mediated wound healing in skin.

Collectively, our findings demonstrate that supersulfide is present in the human skin and plays an important role in regulating keratinocyte migration. Based on our data, we propose that supersulfide in the skin represents a new mechanism of re-epithelialization and may serve as a therapeutic target for skin wounds.

## Funding

This study did not receive any specific grants from funding agencies in the public, commercial, or non-profit sectors.

## Conflict of Interest

The authors declare no competing interests.

## Data Availability Statement

All data are contained within this manuscript.

## Author Contributions

Conceptualization: K. Kunihiro.

Investigation: K. Kunihiro.

Supervision: K. Sano.

Writing–original draft: K. Kunihiro.

## Ethical Approval

The human experiments were approved by the ethics committee of KAC for human tissue products.

## Abbreviations

Akt: protein kinase B
CARS2: cysteinyl-tRNA synthetase 2
CXCL: C-X-C motif chemokine ligand
DAPI: 4',6-diamidino-2-phenylindole
ERK: extracellular signal-regulated kinase
GAPDH: glyceraldehyde-3-phosphate dehydrogenase
MAPK: mitogen-activated protein kinase
MMP: matrix metalloproteinase
PCR: polymerase chain reaction
PI3K: phosphatidylinositol-3 kinase
siRNA: small interfering RNA
SSP4: sulfane sulfur probe 4
