# Supplementary material for: Supersulfides regulate cell migration in human skin keratinocytes
Source: Cell Struct Funct. 2025 Jul 30;50(2):169–82. doi: 10.1247/csf.25018 (PMC12967524; doi:10.1247/csf.25018)
Supplement: Supplementary file 1 — Supplementary Materials [file csf_50_25018_1.zip › 50_25018_Supplemental_Data.docx]

## Supplemental Data

### Table. S1 Quantification of supersulfides in human skin tissue and keratinocytes.

| Supersulfides | Skin tissiue  (pmol/mg wet weight) | Keratinocytes  (pmol/mg protein) |
| --- | --- | --- |
| CysSSH | 2.00 | 6.5 |
| CysSSSH | 0.56 | 16.8 |
| GSSH | 0.02 | 84.9 |
| GSSSH | 0.04 | 14.9 |
| GSSG | 14.0 | 2024.7 |
| GSSSG | 0.98 | 11.1 |

### Fig. S1 Cell viability 24–72 h after transfection of scRNA or siRNA was assessed using a calcein-AM assay.

Cell viability was calculated relative to that of normal cells. Data represent the mean ± SD.


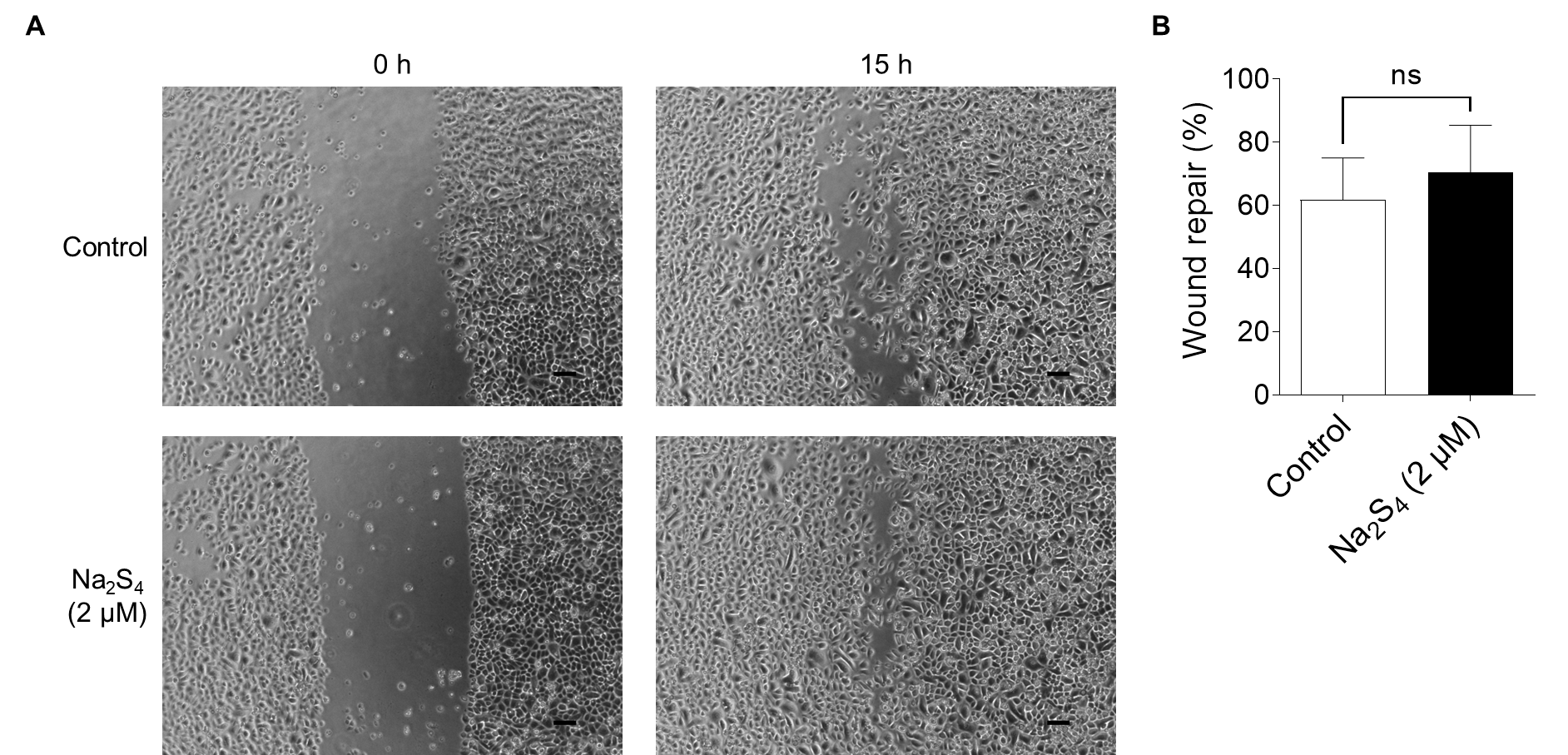


### Fig. S2 Effect of Na_2_S_4_ on cell migration in keratinocytes.

Na_2_S_4_ was added to keratinocytes after scratching. Images were taken by time-lapse every hour for 15 h (A). The scale bar indicates 100 μm. (B) Quantification of the percentage of wound repair. Data represent mean ± SD.
